# Regulation of male sex determination: genital ridge formation and *Sry* activation in mice

**DOI:** 10.1007/s00018-014-1703-3

**Published:** 2014-08-20

**Authors:** Satomi S. Tanaka, Ryuichi Nishinakamura

**Affiliations:** Department of Kidney Development, Institute of Molecular Embryology and Genetics, Kumamoto University, 2-2-1 Honjo, Kumamoto, 860-0811 Japan

**Keywords:** Six1, Six4, Sox9, Transcriptional network, Nr5a1/Ad4BP/Sf1

## Abstract

Sex determination is essential for the sexual reproduction to generate the next generation by the formation of functional male or female gametes. In mammals, primary sex determination is commenced by the presence or absence of the Y chromosome, which controls the fate of the gonadal primordium. The somatic precursor of gonads, the genital ridge is formed at the mid-gestation stage and gives rise to one of two organs, a testis or an ovary. The fate of the genital ridge, which is governed by the differentiation of somatic cells into Sertoli cells in the testes or granulosa cells in the ovaries, further determines the sex of an individual and their germ cells. Mutation studies in human patients with disorders of sex development and mouse models have revealed factors that are involved in mammalian sex determination. In most of mammals, a single genetic trigger, the Y-linked gene *Sry* (sex determination region on Y chromosome), regulates testicular differentiation. Despite identification of *Sry* in 1990, precise mechanisms underlying the sex determination of bipotential genital ridges are still largely unknown. Here, we review the recent progress that has provided new insights into the mechanisms underlying genital ridge formation as well as the regulation of *Sry* expression and its functions in male sex determination of mice.

## Introduction

**Fig. 1:**
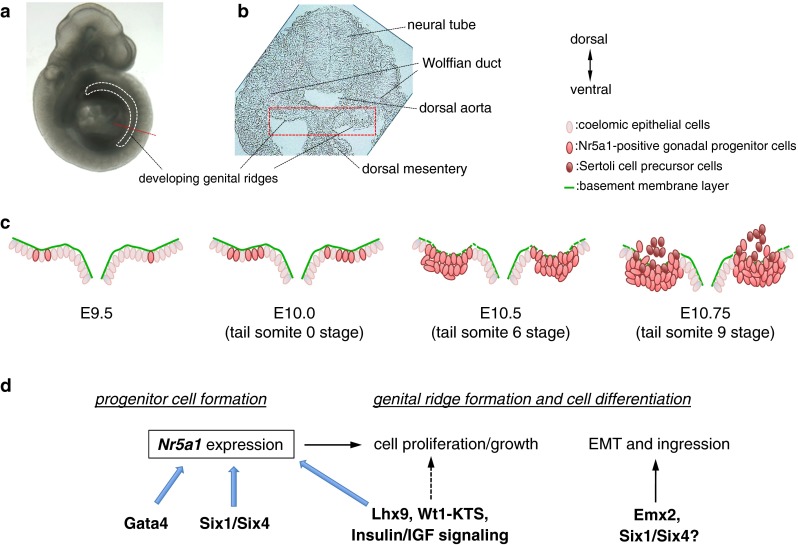
Formation of genital ridges. In mouse embryos, genital ridges are formed on the ventral surface of the mesonephros as paired thickenings of the epithelial layer, which is accompanied by proliferation of the coelomic epithelium from the anterior portion. **a** Embryonic day (E) 10 mouse embryo. *White dashed line* indicates the location of developing genital ridges. *Red dashed line* indicates the position of the section in (**b**). **b** Transverse section of developing genital ridges, representing the dorsal to the *top* and the ventral to the *bottom*. **c** Schematic illustrations of genital ridge formation. The *rectangle* in **b** outlines the approximate position in **c**. Some Nr5a1 (also known as Ad4BP/Sf1)-positive gonadal progenitor cells are formed in the E9.5 coelomic epithelium. The number of Nr5a1-positive cells increases at E10.0 (around the 0 tail somite stage), and multilayered Nr5a1-positive cells are expanded at E10.5 (around the 6-tail somite stage) in the coelomic epithelium. Thereafter, Nr5a1-positive progenitor cells migrate into the dorsal inner mesenchyme region through the basement membrane layer to form the genital ridge primordium (E11.75, around the 9-tail somite stage). In XY gonads, a proportion of Nr5a1-positive daughter cells derived from the coelomic epithelium express *Sry* to become Sertoli cell precursors. **d** Scheme for the molecular network that regulates formation and development of Nr5a1-positive gonadal progenitor cells. Genital ridge formation begins from the anterior part of the coelomic epithelium, which is accompanied by Gata4 and subsequent *Nr5a1* expression. Six1 and Six4 directly transactivate *Nr5a1* in gonadal progenitor cells of the coelomic epithelium. Lhx9, Wt1−KTS, and insulin/insulin-like growth factor (IGF) signaling activity are required to promote gonadal progenitor cell proliferation and form the bipotential genital ridges, which are accompanied by *Nr5a1* upregulation. Emx2 and possibly Six1 and Six4 contribute to regulation of the epithelial-to-mesenchymal transition (EMT) and subsequent ingression of the progenitor cells

The genital ridge is the somatic precursor of gonads in both sexes. This is a unique primordium in organ formation because of its bipotential nature. A single primordium gives rise to one of two organs, a testis or an ovary. The formation of genital ridges begin on the ventral surface of the mesonephros as paired thickenings of the epithelial layer at around embryonic day (E) 9.5 in mouse embryos (Fig. 1). This occurrence is accompanied by proliferation of the coelomic epithelium that gives rise to the somatic lineage precursors of the gonad. When the coelomic epithelium proliferates, the underlying basement membrane becomes fragmented to facilitate the migration of coelomic epithelial cells into the dorsal inner mesenchyme region through the basement membrane layer to form genital ridges (Fig. 1).

The genital ridge is composed of somatic cell lineages and germ cells. However, these two lineages are formed at different developmental stages and positions in the embryo. Progenitor cell formation of germ cells begins with activation of PR domain zinc finger protein (*Prdm*) 1 (also known as *Blimp1*) in a subset of epiblast cells in the proximal region of the pre-gastrulation mouse embryo at around E6.25. Progenitor cells form a cellular cluster and express *Prdm1* along with interferon-induced transmembrane protein (*Ifitm*) 3 (also known as *mil*-*1/fragilis*) at the posterior end of the streak stage embryo at around E6.75. At E7.25, primordial germ cells (PGCs) are specified in the progenitor cell cluster and then translocate from the mesoderm to the endoderm. Thereafter, PGCs are incorporated into the hindgut invagination and then distributed along the length of the embryonic gut. PGCs further migrate through the dorsal mesentery and settle into the genital ridge at around E10.0 [1–11]. After PGC colonization, the decision occurs for the bipotential gonad to develop as either a testis or an ovary. The fate of the gonad is determined by differentiation of somatic cells into Sertoli cells or granulosa cells. Sertoli cells in XY gonads and granulosa cells in XX gonads are the supporting cells that interact with and nurture the germ cells. Therefore, sex determination is essential for sexual reproduction to produce the next generation by the formation of functional male or female gametes. Furthermore, gonadal somatic cells play crucial roles in germ cell development in the gonads of both sexes through their cellular interactions, but the precise mechanisms are unclear.

There are widely diverse systems of sex determination in the animal kingdom. In mammalian sex determination, expression of the Y-linked gene *Sry* (sex determination region on Y chromosome) shifts the bipotential embryonic gonad toward a testicular fate [12–14]. This Sry system appears to be unique to mammals, although the absence of *Sry* has been reported in some species of eutherian mammals [15]. The primary function of Sry is to induce differentiation of pre-Sertoli cells, which is essential for testis differentiation of the bipotential gonad. The fate of the embryonic gonad further determines the sex of an individual and the germ cells. In testes, germ cells differentiate into sperms, whereas in ovaries, germ cells differentiate into oocytes. These male and female gametes combine and generate the next generation by mixing their genetic information.

Therefore, formation of the genital ridge, sex determination of bipotential gonads, and subsequent testicular or ovarian differentiation are critical steps not only to establish sex of an individual, but also to generate the next generation by the formation of functional male or female gametes.

In human patients, disorders of sex development (DSD) are congenital conditions characterized by atypical development of chromosomal, gonadal, or anatomical sex (for a review [16, 17]). It is estimated that up to 2 % of all live births have DSD [18]. Mutation studies in human patients with DSD and mouse models have revealed factors that are involved in sex development. Most of the factors influencing sex determination are transcriptional regulators, whereas factors influencing sex differentiation are frequently related to hormonal signaling. In particular, mouse models employing targeted mutagenesis and transgenesis have contributed greatly to our understanding of gene functions and the transcriptional/signaling networks in sex development (for reviews [19–26]). Thus far, molecular mechanisms underlying genital ridge formation and *Sry* activation in male sex determination are poorly understood, unlike the subsequent testicular or ovarian differentiation. However, recent studies in mouse models have provided new insights into these critical steps. In this review, we mainly focus on the early stages of genital ridge formation and *Sry* activation during male sex determination in mice.

## Formation of the genital ridge

### Overview of genital ridge formation and development

The formation of genital ridges begins on the ventral surface of the mesonephros as paired thickenings of the epithelial layer, which is accompanied by proliferation of the coelomic epithelium at around E9.5 in mouse embryos (Fig. 1). Cell fate mapping analyses revealed that coelomic epithelial cells give rise to somatic lineages of the bipotential gonad. Some coelomic epithelial cells proliferate, undergo epithelial-to-mesenchymal transition (EMT), and migrate into the dorsal inner mesenchyme region to form genital ridges [27–29]. Mutant mouse analyses have shown that several factors, especially some key transcription factors, are involved in the formation and development of genital ridges. The key genes involved in genital ridge formation are outlined in “Box 1”.

Impaired gonadal formation in most mutant mouse embryos is associated with downregulation or ectopic upregulation of the orphan nuclear receptor *Nr5a1* (also known as *Ad4BP/Sf1*). Embryos lacking the LIM homeobox protein *Lhx9* (*Lhx9* ^−*/*−^) exhibit impaired gonad formation accompanied by a significant reduction of *Nr5a1* expression [30]. Embryos lacking the zinc finger transcription factor *Wt1* fail to develop kidneys and gonads [31]. An isoform of *Wt1* lacking an additional three amino acids (lysine, threonine, and serine) (*Wt1* *−* *KTS*) is also essential for the formation and development of the bipotential gonad [32]. Wt1−KTS binds to the *Nr5a1* promoter and activates its expression in cooperation with Lhx9 [33]. In embryos with conditional inactivation of the GATA zinc finger transcription factor *Gata4* after E8.75, impaired genital ridge formation is corroborated by the absence of *Lhx9* and *Nr5a1* expression [34]. Embryos lacking the chromatin modification and remodeling factor *Cbx2* (also known as *M33*) (*Cbx2* ^−*/*−^) show gonadal growth defects accompanied by reduced expression of *Lhx9*, *Nr5a1*, and *Gata4* [35, 36]. Furthermore, chromatin immunoprecipitation (ChIP) assays using adrenocortical Y-1 cells show direct binding of Cdx2 to the *Nr5a1* locus [37]. Embryos lacking the insulin/insulin-like growth factor (IGF) signaling pathway show impaired gonadal development accompanied by a decrease in *Nr5a1* expression [38, 39]. Embryos lacking the basic helix–loop–helix transcription factor *Pod1* (*Pod1* ^*lacZ/lacZ*^) are markedly hypoplastic in both XX and XY gonads, which is accompanied by ectopic expansion of the Nr5a1 expression domain in the gonads and mesonephroi [40]. Biochemical approaches further demonstrate that Pod1 transcriptionally represses *Nr5a1* expression [40, 41].

We also found that homeodomain proteins Six1 and Six4 regulate *Nr5a1* expression in genital ridge formation [42]. *Six1* and *Six4* genes belong to the mammalian homolog of the *Drosophila* *sine oculis* homeobox (*Six*) family, which includes six member genes (*Six1* to *Six6*) in the mouse genome. Six1 and Six4 have redundant functions in mouse embryonic development, possibly through transactivation of common target genes, because Six1 and Six4 bind to a common binding site (MEF3 site) for transactivation (for reviews [43, 44]). *Six1* and *Six4* double-mutant (*Six1* ^−*/*−^ *; Six4* ^−*/*−^), but not *Six1* ^−*/*−^ or *Six4* ^−*/*−^ single-mutant mouse embryos, have smaller gonads and adrenal glands than those of their control counterparts [42, 45]. This abnormality is accompanied by a significant reduction in the expression of *Nr5a1*, but not *Gata4* or other genes involved in gonadal formation. Reporter and ChIP assays have further shown that Six1 and Six4 transactivate *Nr5a1* expression through the MEF3 site at the 5′ flanking region of *Nr5a1* in the M15 mouse mesonephric cell line [42].

In addition, the EMT and subsequent ingression of gonadal progenitor cells are critical steps for genital ridge formation, but precise mechanisms underlying the regulation of EMT remain unclear. The paired-like homeobox protein *Emx2* has been implicated in the maintenance of epithelial polarity and the EMT and subsequent ingression of gonadal progenitor cells, possibly through the suppression of EGF receptor (*Egfr*) expression [28]. Although *Emx2* expression is not downregulated, *Six1* ^−*/*−^ *; Six4* ^−*/*−^ genital ridges show delayed/reduced EMT and subsequent ingression of gonadal progenitor cells [42]. Ectopic expression of human *SIX1* in the mammary gland epithelium of adult mice has been reported to induce tumors. *SIX1* misexpression facilitates expansion of the mammary epithelial stem/progenitor cell pool and induces mammary tumors that undergo EMT [46]. These observations suggest that Six1 and Six4 are also implicated in regulation of the EMT and subsequent ingression of gonadal progenitor cells.

Collectively, these findings indicate that Nr5a1 is a critical factor for the formation and development of gonadal precursor cells (Fig. 1). At the initial stage of genital ridge formation, Gata4, Six1, and Six4 contribute to the formation of *Nr5a1*-positive gonadal progenitor cells in the coelomic epithelium. Lhx9, Wt1−KTS, and insulin/IGF signaling activity are also required for *Nr5a1* expression and promotion of gonadal progenitor cell proliferation in bipotential genital ridges. In addition, Emx2 and possibly Six1 and Six4 contribute to the regulation of EMT and subsequent ingression of progenitor cells (Fig. 1). After the ingression, Cbx2 and probably Pod1 contribute to *Nr5a1* expression and progenitor cell growth and/or differentiation in the bipotential gonad.

### Potential functions of Nr5a1 in genital ridge formation

It has been proposed that Nr5a1 acts dose dependently. Nr5a1-positive progenitor cells in the coelomic epithelium give rise to the somatic lineages of gonads and adrenal glands. In *Nr5a1* ^+*/*−^ mouse embryos, their adrenal glands are underdeveloped and show reduced cellular proliferation [47]. Compound mutant studies in *Six1* ^+*/*−^, *Six4* ^+*/*−^, and *Nr5a1* ^+*/*−^ embryos also demonstrate impaired formation of gonadal progenitor cells, which is dependent on the *Nr5a1* expression level [42]. Embryos with homozygous deletion of *Nr5a1* (*Nr5a1* ^−*/*−^) exhibit regression of the gonads by E12.5 with apoptosis of gonadal somatic cells [48–50]. In contrast, ectopic expansion of the Nr5a1 expression domain in *Pod1* ^*lacZ/lacZ*^ gonads is accompanied by an increase in the number of fetal Leydig cells [40]. In addition, *Nr5a1* overexpression in *Nr5a1* ^−*/*−^ mice rescues the impaired gonad and spleen development, but not the impaired adrenal gland development. This difference in rescue effects might be dependent in part on the differential levels of *Nr5a1* expression among tissues and differential sensitivities to the gene dosage [51].

Nr5a1 plays critical roles in the activation of a set of genes involved in steroidogenesis, such as *Cyp17a1* and *3β*-*Hsd* in Leydig cells. Indeed, *Nr5a1* was first identified as a gene encoding a common transactivating factor of steroidogenic genes [52–54]. Nr5a1 also plays important roles in a variety of physiological activities (for reviews [55, 56]). It could be postulated that Nr5a1 modulates the expression of target gene sets that are implicated in various physiological activities, including metabolism and stimulation of cell proliferation, differentiation, and survival, which are essential for gonadal development. This hypothesis is supported by the impaired gonad and adrenal gland formation in embryos lacking the insulin/IGF signaling pathway. The insulin/IGF signaling pathway is known to modulate a variety of physiological activities (for a review [57]). Mouse embryos lacking the insulin/IGF signaling pathway show reduced proliferation of gonadal and adrenal progenitor cells, which is accompanied by downregulation of hundreds of genes including *Nr5a1* [39]. Thus, reduced Nr5a1 activity might impair the physiological activities of the progenitor cells, resulting in impaired gonad and adrenal gland formation.

At the later stage, Nr5a1 regulates the expression of key genes that are crucial for testicular differentiation in XY gonad development, such as Sry-related HMG box 9 (*Sox9*) and Müllerian inhibitory substance [*MIS*, also known as anti-Müllerian hormone (*Amh*)] [58, 59].

### Initiation of genital ridge formation and *Nr5a1* upregulation

A subpopulation of coelomic epithelial cells that express *Gata4* and *Nr5a1* are thought to be the initial population that gives rise to the somatic lineages of the genital ridge. Thus far, the upstream regulator(s) of *Gata4* in genital ridge formation are unknown. In contrast, as described above, several upstream regulators of *Nr5a1* have been reported by analyses of mutant mouse embryos (“Box 1”). *Nr5a1* expression is regulated through several lineage-specific enhancers such as the fetal Leydig cell-specific enhancer in the embryonic gonad [60]. Therefore, combinations of upstream regulatory factors may facilitate the initiation and maintenance of *Nr5a1* expression in gonadal progenitors and specific lineages in developing gonads. At the onset of *Nr5a1* expression, the coelomic epithelium expresses Six1, Six4, and Gata4, suggesting that these factors may preferentially contribute to the initiation of *Nr5a1* expression in gonadal progenitor cells.

Genital ridges are extremely long and narrow structures along the anterior–posterior (A–P) axis. Recently, Hu et al. [34] reported that the anterior part of the monolayered coelomic epithelium expresses Gata4 at the onset of genital ridge formation (E9.25, 26–27 total somite stage). The Gata4 expression pattern in the coelomic epithelium, which precedes thickening and progresses in an A–P direction, is well correlated with the A–P progression of genital ridge formation [34]. Soon after (E9.5, 0 tail somite stage), the coelomic epithelium begins to express Nr5a1 and forms the thickened (multilayered) structure [42, 61]. Expression of Nr5a1 is also extended in the A–P direction following the Gata4 expression pattern in the coelomic epithelium. At the anterior region, the coelomic epithelium is developmentally more advanced than that at the posterior region, in which the E10.4 (6 tail somite stage) anterior but not posterior coelomic epithelium has already become more than one layer of cells [34]. These findings suggest that the formation of the extremely long and narrow genital ridge begins from the anterior part of the coelomic epithelium, which is accompanied by Gata4 and subsequent Nr5a1 expression. The Gata4 upregulation pattern during genital ridge formation appears to be closely correlated with Nr5a1 rather than Six1 or Six4. However, during the initial growth of gonadal precursor cells, Six1 and Six4 expression is colocalized with high Nr5a1 expression and they are considered to directly transactivate *Nr5a1* in the coelomic epithelial cells at E11.0 (12 tail somite stage). It is likely that Gata4 facilitates the initial activation of *Nr5a1*, while Six1 and Six4 may contribute to the maintenance of high *Nr5a1* expression in the progenitors. Nonetheless, a proportion of the cells that express Gata4, Six1, or Six4 appear to become *Nr5a1*-positive progenitor cells in the coelomic epithelium. Further studies are required to uncover the precise mechanisms underlying the restricted upregulation of *Nr5a1* in the subpopulation of coelomic epithelial cells. Furthermore, identification of the upstream regulator of *Gata4*, *Six1*, and *Six4* remains to be elucidated in the coelomic epithelium.

## *Sry* expression and subsequent *Sox9* upregulation

### Overview of sex determination in bipotential gonads

**Fig. 2:**
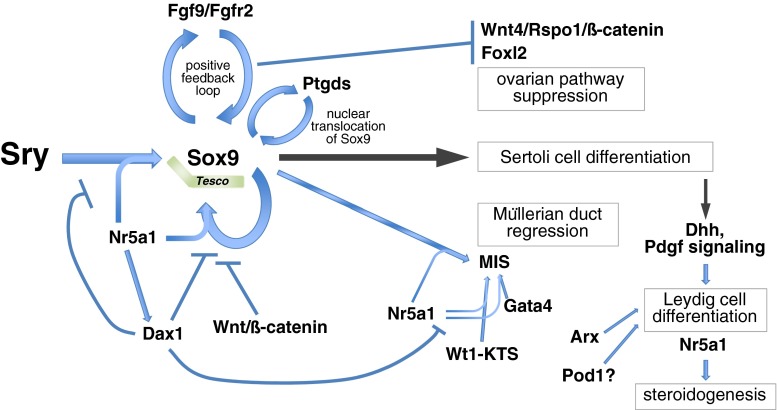
Sry and the transcriptional network that governs testis determination. In mice, expression of a single genetic trigger, the Y-linked gene *Sry*, induces differentiation of pre-Sertoli cells. Sry directly transactivates *Sox9* through the core element of the testis-specific enhancer region of *Sox9* (*Tesco*) together with Nr5a1. Sox9 itself also contributes to the maintenance of *Sox9* expression through *Tesco* together with Nr5a1. Excess Dax1 interferes with *Sox9* upregulation, likely through inhibition of the binding of Nr5a1/Sry or Nr5a1/Sox9 proteins to *Tesco.* Although Dax1 interferes with the activity of Nr5a1, *Dax1* expression depends on Nr5a1 activity. Sox9 upregulates *Fgf9* expression, and FGF9 in turn establishes the Sox9–FGF9 positive feedback loop through FGF receptor 2 (FGFR2), which maintains a high level of *Sox9* expression. The Sox9–FGF9 positive feedback loop also acts to suppress ovary-specific WNT4/R-spondin1(Rspo1)/β-catenin signaling activity. *Sox9* expression also interferes with upregulation of the ovarian gene *Foxl2*. In addition, Sox9 upregulates *Ptgds*, and the signaling activity of Ptgds promotes nuclear translocation of Sox9 to facilitate Sertoli cell differentiation. Together with Nr5a1, Sox9 regulates activation of *MIS* that promotes regression of Müllerian ducts. *MIS* is also regulated synergistically by Nr5a1 and Wt1−KTS, as well as Gata4. Sertoli cells express Dhh that is required for specification of the fetal Leydig cell fate. Pdgf secreted by Sertoli cells is also required for fetal Leydig cell differentiation. Arx and probably Pod1 are involved in regulation of Leydig cell differentiation. Male steroid hormones are synthesized by Leydig cells, which is mainly regulated by *Nr5a1*

The fate of the embryonic gonad is determined by differentiation of somatic cells into Sertoli or granulosa cells. In bipotential gonads, the formation of Sertoli cells promotes the testicular differentiation program, whereas formation of granulosa cells promotes the ovarian differentiation program. In most of mammalian sex determination, expression of the Y-linked gene *Sry* shifts the bipotential embryonic gonad toward a testicular fate [12–14]. The primary function of Sry is to induce differentiation of pre-Sertoli cells, which is essential for testis differentiation of the bipotential gonad (Fig. 2). *Sry* shows a strictly controlled and limited spatiotemporal expression pattern in the precursors of Sertoli cells. In mouse genital ridges, *Sry* is first expressed at around E11.0 (12 tail somite stage), reaches peak levels of expression at E11.5 (18 tail somite stage), and is extinguished shortly after E12.5 (30 tail somite stage). Expression of *Sry* begins in the central region of genital ridges and then extends to the anterior and posterior poles. Thereafter, *Sry* expression extinguishes in the anterior and central regions, and becomes restricted to the posterior region before it completely disappears in the genital ridges. At about 4 h after initiation of *Sry* expression, *Sox9* is upregulated in Sertoli cell precursors [62–69]. Transgenic mouse analyses have demonstrated that the expression of either *Sry* or *Sox9* in the bipotential gonad is sufficient to induce the male developmental program [13, 70–72].

### Roles of Sry and Sox9 in testis determination

Sry and Sox9 are members of the Sox family of developmental transcription factors that contain an amino acid motif known as the HMG domain (for reviews [73, 74]). This HMG motif enables Sox family proteins to bind to the DNA consensus sequence (A/T)ACAA(T/A) with high affinity [75]. Most SRY mutations found in human patients showing male-to-female sex reversal affect the ability of SRY to bind and bend DNA [76–80]. SRY mutation analysis of its C-terminal domain suggests that the SRY C-terminal domain may contribute to the conformation of SRY and a change in conformation may influence SRY functions [81]. There is a nuclear localization signal (NLS) at the N-terminal end of the SRY HMG box, and SRY mutations in this NLS result in a reduction of nuclear importation, which partially explains some cases of human sex reversal [82, 83].

A *Sox9* transgene has been found to promote the testicular differentiation program instead of *Sry* [70–72]. Thus, the essential function of Sry in testis determination may be upregulation of *Sox9* only. Another possibility is that the *Sox9* transgene product activates not only endogenous *Sox9*, but also other Sry target genes that are required to induce testicular development. Therefore, *Sry* is dispensable for testicular development in *Sox9* transgenic embryos. Several genes have been demonstrated as prospective downstream targets of Sry and/or Sox9 by ChIP assays, such as *Pod1*, the secreted growth factor neurotrophin 3 (*Ntf3*), and secreted glycoprotein cerebellin precursor 4 (*Cbln4*) [84–87]. However, there is no direct evidence of the possible involvement of these genes in the initial testis determination (Sertoli cell differentiation) that might be regulated by Sry, but not Sox9, in XY gonads [40, 88, 89], except for *Sox9* [90, 91]. Nonetheless, further studies will be required to determine the functions of Sry in testis determination.

### Upstream regulatory factors of *Sry*

Although a *Sox9* transgene promotes the testicular differentiation program in bipotential gonads, primary sex determination in mammals is commenced by the presence or absence of the Y chromosome. *Sox9* is located on an autosome (chromosome 11 in the mouse genome), whereas *Sry* is on the Y chromosome. Therefore, the Y-linked gene *Sry* is the single genetic trigger that determines testis formation in the bipotential gonad of XY mammals. A 14.6 kb *Sry* transgene construct can mimic endogenous *Sry* expression in transgenic mouse embryos [62]. However, this construct lacks the cis-acting regulatory element that is necessary for transcriptional silencing after E 12.5. Furthermore, there are no reports of specific *cis*-acting regulatory elements that are implicated in transcriptional activation of *Sry* in vivo. In vitro biochemical analyses have demonstrated that WT1, NR5A1, SOX9, GATA4, and SP1 bind to and transactivate human or pig *SRY* promoters [92–96]. There is limited knowledge of the regulation of *Sry* expression in vivo. Therefore, genetic inactivation of genes, especially genes encoding some key transcription factors, results in reduced *Sry* expression and a sex reversal phenotype. The key genes involved in *Sry* expression are outlined in “Box 2”. For example, although Wt1−KTS binds to the *SRY* promoter region [93], testicular differentiation markers are expressed in a small cluster of cells in mouse embryos that specifically lack *Wt1−KTS* [32]. This finding suggests that Wt1−KTS is unlikely to be required for *Sry* expression in testis determination. In contrast, abolition of the *Wt1*+*KTS* isoform results in reduced *Sry* levels and a sex-reversal phenotype [32]. Wt1+KTS does not transactivate the *Sry* promoter in vitro [93, 96], but is reported to function to increase the levels of unspliced RNA containing either a cellular or viral constitutive transport element and to specifically promote translation of this unspliced RNA [97]. These findings suggest that Wt1+KTS is implicated in the post-transcriptional regulation of *Sry* mRNA in testis determination.

Embryos lacking a gene encoding a zinc finger protein Friend of GATA-2 (*Fog2*, also known as *Zfpm2*) and those containing homozygous mutant alleles of *Gata4* ^*ki*^, which abrogate the interaction of Gata4 with Fog, show reduced *Sry* expression and a sex-reversal phenotype [98]. These findings suggest that the interaction of Gata4 and its co-factor Fog2 is critical for *Sry* activation. We also found that Six1 and Six4 play crucial roles in *Sry* expression by upregulation of *Fog2* in the coelomic epithelium. XY *Six1* ^−*/*−^; *Six4* ^−*/*−^ gonads show remarkable downregulation of *Sry* and subsequent impaired testicular differentiation accompanied by reduced *Fog2* expression. Reporter assays in the M15 mouse mesonephric cell line and ChIP assays using embryonic tissues containing gonads further demonstrated that *Fog2* is a direct target of Six1 and Six4 [42].

Recently, it was reported that stage-specific *Sry* upregulation is mediated by transient activation of Gata4 via its phosphorylation. In a forward genetic screen of mouse homozygous mutants exhibiting consistent XY gonadal sex reversal, Bogani et al. (2009) identified a recessive boygirl (*byg*) mutation. The *byg* mutation is an A to T transversion that introduces a premature stop codon in the gene encoding mitogen-activated protein kinase (*Mapk*) kinase kinase *Map3k4* (also known as *Mekk4*). On the C57BL/6J background, E11.5 *byg/byg* gonads show impaired growth and a dramatic reduction of *Sry* expression. MKK4, a direct target of MAP3K4 and p38 MAPK, is activated in the coelomic region of the E11.5 XY wild-type gonad, suggesting that MAPK signaling may be involved in promoting gonadal somatic cell growth and regulation of *Sry* expression [99]. MAP3K4 interacts with several proteins including members of the growth arrest and DNA damage response protein family [100]. Mice lacking a member of this family, *Gadd45g* (*Gadd45g* ^−*/*−^), also show XY gonadal sex reversal caused by disruption to *Sry* expression [101, 102]. *Gadd45g* and *Map3k4* genetically interact during sex determination, and transgenic overexpression of *Map3k4* rescues gonadal defects in *Gadd45g* ^−*/*−^ embryos. In *Gadd45g* ^−*/*−^ gonads, there is a delay and reduction in *Sry* expression, despite the fact that the *Sry* promoter is demethylated and occupied by active histone marks. Instead, the sex-reversal phenotype in both *Gadd45g* and *Map3k4* mutants is associated with reduced phosphorylation of p38 MAPK and Gata4. Conditional inactivation of the genes encoding *p38α* and *p38ß* *Mapks* also causes embryonic XY gonadal sex reversal due to reduced levels of *Sry* expression. Furthermore, reduced levels of phosphorylated Gata4 are found in both *Gadd45g* and *Map3k4* mutant XY gonads, and Gata4 binds to the *Sry* promoter in vivo in a MAPK-dependent manner [101, 102]. Remarkably, *Gadd45g* shows increased expression at the onset of *Sry* expression in the genital ridges of both sexes. This increased expression of Gadd45g is considered to regulate stage-specific *Sry* expression by interacting with Map3k4 [101, 102]. *Sry* is also expressed in non-gonadal tissues such as dopamine-abundant regions of the brain. It has been recently reported that the *Sry* upregulation pathway in Sertoli cell precursors appears to be conserved in neuronal cells of the brain [103]. Treatment with a dopaminergic toxin, 6-hydroxydopamine, induces an increase of *Gadd45g* expression and activates the Gadd45g–Map3k4–p38 MAPK pathway, resulting in *SRY* upregulation in human male neuroblastoma-derived cell line M17 cells [103].

Histone modification factors are also reported to be involved in *Sry* expression. Mouse embryos lacking the polycomb group gene *Cbx2* show reduced *Sry* expression [35]. However, the genetic interaction between *Cbx2* and *Sry* is unclear. Recently, Kuroki et al. [104] reported that male-to-female sex reversal in mice lacking the histone H3 lysine 9 (H3K9) demethylase *Jmjd1a* (also known as *Tsga/Jhdm2a/Kdm3a*) (*Jmjd1a* ^−*/*−^) is accompanied by reduced expression of *Sry*. *Jmjd1a* ^−*/*−^ mice show abnormal sex differentiation depending on the genetic background. On the CBA genetic background, 88 % of XY *Jmjd1a* ^−*/*−^ mice show abnormal sex differentiation, whereas only 14 % of XY *Jmjd1a* ^−*/*−^ mice on the B6 genetic background show such a phenotype. At E11.5, Jmjd1a is expressed in gonadal somatic and germ cells, but not mesonephric cells. *Jmjd1a* shows the highest expression level among the genes encoding enzymes involved in the maintenance of H3K9 methylation in E11.5 gonadal somatic cells. *Jmjd1a* expression increases from E10.5 and reaches a peak at around E11.5. Interestingly, inactivation of *Jmjd1a* is unlikely to influence the expression of known *Sry* regulators. Instead, Jmjd1a binds to regulatory regions within the *Sry* locus as shown by ChIP assays using purified Nr5a1-positive gonadal somatic cells from E11.5 gonads. Furthermore, inactivation of *Jmjd1a* leads to a significant increase in the levels of H3K9 demethylation (H3K9me2) within the *Sry* locus without changing histone H3 occupancy and the H3K9me2 levels of the *Sox9* locus [104]. Therefore, these findings suggest a crucial role of a histone demethylase in *Sry* expression. It is likely that the H3K9me2 marks may limit the ability of the transcriptional factors (i.e., their accessibility or initiation of transcription) to facilitate the *Sry* upregulation, because *Sry* regulators are considered to be normally present in *Jmjd1a* ^−*/*−^ gonads.

**Fig. 3:**
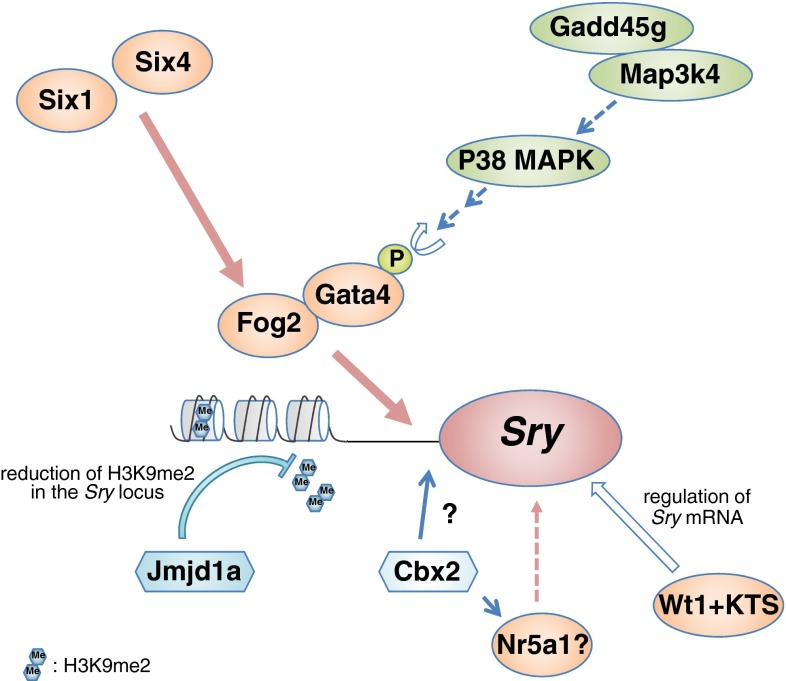
Model for *Sry* upregulation. Before the onset of *Sry* expression, a reduction in the H3K9me2 levels of the *Sry* locus is mediated by stage-specific upregulation of the H3K9 demethylase Jmjd1a, which may allow *Sry* upregulation by transcriptional factors. Interactions of Gata4 with its co-factor Fog2 are critical for *Sry* activation. *Fog2* is upregulated in the coelomic epithelium by Six1 and Six4 before the onset of *Sry* expression. Gata4 is transiently activated by the Gadd45g-Map3k4-p38 MAPK pathway because of the stage-specific *Gadd45g* upregulation. Subsequently, the phosphorylated Gata4 and Fog2 protein complex may bind to the *Sry* promoter and activate *Sry* expression in a stage-specific manner. The Wt1+KTS isoform may contribute to the post-transcriptional regulation of *Sry* mRNA. The polycomb group gene *Cbx2* is required for *Sry* upregulation, but the genetic interaction between *Cbx2* and *Sry* is unclear. In addition, Cbx2 promotes *Nr5a1* upregulation, and Nr5a1 is proposed to be one of the upstream regulators of *Sry*

Collectively, this recent progress has revealed the molecular network that governs *Sry* upregulation (Fig. 3). Before the onset of *Sry* expression, H3K9me2 levels are reduced in the *Sry* locus, which is mediated by stage-specific upregulation of Jmjd1a, allowing initiation of *Sry* expression by the transcriptional factors. *Fog2* expression is also upregulated in the coelomic epithelium by Six1 and Six4 before the onset of *Sry* expression. Gata4 is transiently activated by the Gadd45g–Map3k4–p38 MAPK pathway. Subsequently, the phosphorylated Gata4 and Fog2 protein complex may bind to the *Sry* promoter and activate *Sry* expression in a stage-specific manner. In addition, the Wt1+KTS isoform may contribute to the post-transcriptional regulation of *Sry* mRNA (Fig. 3).

### Upregulation and maintenance of *Sox9* expression

*Sry* shows a strictly controlled and limited spatiotemporal expression pattern in XY gonads. To upregulate *Sox9* and promote subsequent testicular differentiation, the appropriate timing and a sufficient level of *Sry* expression are thought to be required. For example, a mouse strain combination study revealed that the Y chromosome from natural populations of *Mus domesticus* captured in Val Poschiavo, Switzerland (termed Y^POS^), failed to promote normal differentiation of the testis when crossed with a C57BL/6 J background [105]. Some B6-Y^POS^ mice show a range of phenotypes in the impairment of testis development, such as hermaphroditism with ovotestes and complete sex-reversal phenotypes. In B6-Y^POS^ mice, there is a definite delay and likely reduction in *Sry* expression, resulting in impaired *Sox9* expression [106]. Ovotestes of B6-Y^POS^ mice show partial testis cord formation with stable high expression of *Sox9* in the central region, whereas the ovarian somatic cell marker ovarian gene Forkhead box L2 (*Foxl2*) is expressed in the pole regions [107]. Such high expression of *Sox9* in the central region of the gonad is considered to reflect the *Sry* expression that begins in the central region of the gonad. Because of the delay in *Sry* expression, only the central region, but not the pole regions, expresses *Sry* at a sufficient level in the appropriate time window, allowing the cells to upregulate *Sox9* and maintain the high level of expression needed to promote subsequent testicular differentiation.

Hiramatsu et al. [108] established a heat shock-inducible *Sry* transgenic mouse system that allows induction of testis development in cultured XX genital ridges at various time points during development. Using this system, they showed that the ability of *Sry* to determine testis development is limited to a narrow time window of 6 h, approximately from E11.0 to 11.25 (12–15 tail somite stages) [108]. Interestingly, after this critical time period, ectopic *Sry* induction initially induces *Sox9* expression, but the high level of *Sox9* expression is not maintained, resulting in ovarian differentiation. This finding suggests that the action of Sry in the narrow time window to drive testicular development is likely to be limited by maintenance of the high level of *Sox9* expression rather than the initial upregulation of *Sox9* [108].

Furthermore, the presence of an appropriate number of *Sry*-expressing pre-Sertoli cells in the XY gonad might be crucial to maintain the high level of *Sox9* expression and subsequent testicular differentiation. Proliferation of gonadal somatic cells at E11.25–11.5 (a specific 8-h period), which coincides with the initiation of *Sry* expression, is considered to be required to recruit an appropriate number of *Sry*-expressing pre-Sertoli cell precursors from the coelomic epithelium, leading to testis cord formation in developing XY gonads [29, 109]. In addition, FGF9 has been reported to promote the male-specific proliferation of Sertoli cell precursors between E11.0 and 11.5 [110, 111]. Abolition of *Wt1*+*KTS* isoform results in reduced *Sry* levels and produces the male-to-female sex-reversal phenotype [32]. It is accompanied by a decrease in cell proliferation of Sry-expressing cells in the coelomic epithelium, which is rescued by the addition of exogenous FGF9 to the cultured gonad [112]. XY *Six1* ^−*/*−^; *Six4* ^−*/*−^ gonads show impaired growth of gonadal progenitor cells and a remarkable reduction in the number of Sry-expressing cells [42]. In accordance with the center-to-pole *Sry* expression pattern, Sox9-positive cells are initially and predominantly found in the central region and then limited to the pole regions, especially at the posterior region of the gonads, and eventually disappear in XY *Six1* ^−*/*−^; *Six4* ^−*/*−^ gonads. Forced *Sry* transgene expression in XY *Six1* ^−*/*−^; *Six4* ^−*/*−^ (*Six1* ^−*/*−^; *Six4* ^−*/*−^; *Sry* ^*Tg/*+^) gonads rescues the impaired testicular development, which is accompanied by stable high expression of *Sox9*, but not the initial progenitor cell growth. Even in the genital ridge with fewer initial gonadal precursor cells, *Sry* transgene expression might increase the number of *Sry*-expressing cells. Therefore, maintenance of the high expression level of *Sox9* and subsequent testicular differentiation are rescued in XY *Six1* ^−*/*−^; *Six4* ^−*/*−^; *Sry* ^*Tg/*+^ embryos [42].

Sekido and Lovell-Badge [59] revealed that Sry directly transactivates *Sox9* through the 3.2 kb testis-specific enhancer region of *Sox9* (*Tes*) or 1.4 kb of its core element (*Tesco*), together with Nr5a1 in pre-Sertoli precursor cells. ChIP assays show that Sry and Nr5a1 directly bind to several sites within the *Sox9* enhancer region in vivo. Mutations in these sites abolish the *Sox9* enhancer activity in transgenic mice, suggesting that Sry and Nr5a1 synergistically upregulate *Sox9* enhancer activity [59]. Sry may contribute to the initial upregulation of *Sox9*, but not its maintenance at later stages, because *Sry* shows transient upregulation at around E11.5 and then disappears by E12.5 in genital ridges. Alternatively, Sox9 itself may contribute to the maintenance of *Sox9* expression through the *Tes* together with Nr5a1 [59] (Fig. 2). In addition, an excess amount of the X-linked orphan nuclear hormone receptor *Dax1* (also known as *Nr0b1*) causes an XY ovotesticular disorder of sex development. Excess Dax1 interferes with *Sox9* upregulation by likely inhibiting Nr5a1/Sry or Nr5a1/Sox9 protein binding to the testis-specific enhancer region of *Sox9* [113]. Although Dax1 interferes with the activity of Nr5a1 in *Sox9* upregulation, Dax1 expression depends on Nr5a1 activity [114] (Fig. 2). Although mouse *Tes* shows testis-specific enhancer activity [59], human *TES* is unlikely to show such activity in transgenic mice, and mutations have not been identified in human *TES*, which cause DSD (for a review [24]). It suggests that there might be uncharacterized *SOX9* regulatory elements in addition to *TES*. It has been reported that the regulatory region of *SOX9* spans more than 2.5 Mb upstream and downstream of the *SOX9* open reading frame [91, 115]. In addition, a dominant insertional mutation, *Odsex* (*Ods*), in which XX mice carrying a 150 kb deletion (approximately 1 Mb upstream of *Sox9*) develop as XX males lacking *Sry*, is accompanied by *Sox9* upregulation [70]. Recently, a noncoding genomic region of the *Sox9* promoter has been reported to regulate sex determination [116]. In B6-Y^POS^, the presence of a 55 Mb congenic region on chromosome 11, a flanking region of *Sox9*, is known to protect against B6-Y^POS^ sex reversal in a dose-dependent manner. Arboleda et al. [116] further demonstrated that a 1.62 Mb congenic region of the *Sox9* promoter, which is likely derived from the semi-inbred strain POSA, protects against B6-Y^POS^ sex reversal and promotes *Sox9* expression, thereby driving testis development within the B6-Y^POS^ background. Further analyses of mutations to identify the novel testis-specific enhancer element of *SOX9* will be needed in human patients with DSD.

*Sox9* is also reported to be upregulated in a transgenic mouse overexpressing *Sox3* [117]. *Sox3* is an X-linked gene and shows high sequence similarity with *Sry*. *Sox3* is not expressed in male or female developing gonads, and loss-of-function mutations in *Sox3* do not affect sex determination in humans or mice. However, in the *Sox3* overexpressing transgenic mouse embryo, *Sox3* shows ectopic expression in bipotential gonads and induces *Sox9* upregulation, thereby driving female-to-male sex reversal [117]. Three 46, XX DSD patients have also been identified with genomic rearrangements related to *SOX3*. Two of them are duplications including *SOX3*, and the other is a deletion of the putative upstream regulatory region of *SOX3* [117]. Thus, SOX3 and SRY are considered to be functionally interchangeable to upregulate *SOX9* expression in testis determination. This finding also supports the hypothesis that the Y-linked gene *SRY* may have evolved from the X-linked gene *SOX3* (for a review [118]). *Sox9* is also known to be upregulated in a transgenic mouse overexpressing another *Sox* family gene, *Sox10*, thereby driving female-to-male sex reversal [119]. *Sox9*, *Sox10*, and *Sox8* are *SoxE* family genes. *Sox8* and *Sox10* are upregulated in the XY developing gonad shortly after *Sox9* expression [119, 120]. Although neither inactivation of *Sox8* nor *Sox10* in mice results in abnormal sexual development [119, 121], double-mutant mouse studies of *Sox8* and *Sox9* imply that they have functional redundancy in testicular differentiation [122, 123]. In addition, a duplication in the region encompassing *SOX10*, among a number of other genes, has been identified in human 46,XX patients with DSD [124, 125]. Collectively, not only the misexpression of *SRY*, but also *SOX* family genes is considered to promote testicular differentiation by upregulation of *SOX9* or in place of *SOX9* expression in DSD patients.

## Testicular differentiation of the gonad after *Sox9* upregulation

### Overview of sex differentiation of gonads

During the past few decades, we have gained considerable knowledge of the regulatory gene network in testicular differentiation promoted by Sox9 (for reviews, [19–26]). Sox9 directly or indirectly upregulates *Fgf9* expression, and FGF9 in turn upregulates *Sox9* expression [126]. Therefore, *Sox9* is first upregulated by transient expression of *Sry* in pre-Sertoli cells, and then the Sox9–FGF9 positive feedback loop maintains the high level of *Sox9* expression during testicular differentiation of XY gonads (Fig. 2). Mice lacking FGF receptor 2 (*Fgfr2*) show partial XY sex reversal, which phenocopies *Fgf9* mutants, suggesting that FGF9 signaling through FGFR2 is required for testicular development [127, 128]. FGF9 is also known to promote the survival of germ cells and prevents them from entering meiosis [129, 130]. Sox9 also binds directly to the promoter of *Ptgds* encoding prostaglandin D2 synthase to induce upregulation, and its signaling activity promotes nuclear translocation of Sox9 to facilitate Sertoli cell differentiation [69, 131, 132]. Together with Nr5a1, Sox9 regulates the activation of *MIS* that promotes regression of Müllerian ducts [58]. *MIS* is also regulated synergistically by Nr5a1 and Wt1−KTS, as well as Gata4, while Dax1 antagonizes these synergistic effects [133–135] (Fig. 2).

The antagonism between testicular and ovarian genes is known to regulate sex differentiation of the gonad (Fig. 2). In XY gonads, the testis-specific Sox9–FGF9 positive feedback loop acts to suppress ovarian gene expression, leading to promotion of testicular differentiation. In contrast, ovary-specific canonical WNT signaling represses the testis-specific Sox9–FGF9 positive feedback loop in XX gonads, enabling commencement of ovarian differentiation. For example, conditional inactivation of *Sox9* in XY embryonic gonads causes upregulation of ovarian gene *Foxl2* [136]. Conversely, XX embryonic gonads lacking the ovarian gene *Wnt4* are partially masculinized with transient *Sox9* activation. Ovary-specific WNT4/R-spondin1 (Roof plate-specific Spondin 1, Rspo1)/β-catenin signaling represses the testis-specific Sox9–FGF9 positive feedback loop during ovarian differentiation of XX gonads [126, 137–139]. Furthermore, ectopic activation of WNT/β-catenin signaling in XY gonads leads to the loss of Nr5a1 binding to the *Sox9* enhancer region, thereby inhibiting Sertoli cell differentiation [140] (Fig. 2). Remarkably, co-expression of testis-specific Sox9 and ovary-specific Foxl2 has never been found in the same cell, even in a sex-disordered gonad. This observation is the result of the antagonism between testicular and ovarian genes, which regulates sex differentiation of the supporting cell lineage in the gonads.

As described above, the identification of several key genes that regulate sex determination has facilitated our understanding of the regulatory gene network in testicular differentiation. However, our current knowledge still cannot fully explain some cases of sexual development disorders. It is likely that the sexual fate decision in the developing gonad depends on a complex network of interacting factors that converge at a critical threshold. Munger et al. [141, 142] has performed comprehensive analyses of expression quantitative trait loci to elucidate the transcriptional network underlying sex determination. This approach identified autosomal regions that control the expression of many sex-related genes such as *Sry* and *Sox9* [141]. Furthermore, gene-silencing analyses of candidate genes revealed that Lim-domain only 4 (*Lmo4*) is a novel regulator of sex determination upstream of *Nr5a1*, *Sox9*, *Fgf9*, and *Col9a3* [142]. Further comprehensive approaches will be needed to elucidate the regulatory gene network that governs testicular differentiation.

### Cell lineage derivation in gonads

#### Supporting cell lineages (Sertoli cells and granulosa cells)

During genital ridge formation, the first population of somatic cell progenitors from the coelomic epithelium migrates mediodorsally to form the bipotential gonad. In XY gonads, some of the Nr5a1-positive daughter cells derived from the coelomic epithelium express *Sry* to become Sertoli cell precursors [27, 29, 62, 63, 68]. This ability of the coelomic epithelium to give rise to Sertoli cells is developmentally regulated by E10.5 (8 tail somite stage). When the cells are labeled by the fluorescent lipophilic dye at E11.5 (18–20 tail somite stages), the coelomic epithelial cells no longer become Sertoli cells. Instead, the coelomic epithelial cells that migrate into the gonad remain outside of the testis cords and become interstitial cells [27]. During genital ridge formation at around E10.0–11.5, two kinds of Nr5a1-positive cell populations, Nr5a1^high^ and Nr5a1^low^, appear to be in the coelomic epithelium and the mediodorsal region where genital ridges are formed [42]. Because Nr5a1 is known to act dose dependently, the differential expression level of Nr5a1 in progenitors may also be associated with cell fate commitment to the Sertoli cell lineage.

At around E12.5, there is drastic reorganization of XY gonads, leading to a significant difference in the morphologies of the testis and ovary. In XY gonads, Sertoli cells polarize and aggregate around germ cells to form the tubular testis cord. The testis cord is composed of Sertoli and germ cells layered by peritubular myoid cells. Sertoli cells interact with and support the growth and differentiation of germ cells during gametogenesis. Sertoli cells express *Cyp26b1* encoding the P450 catabolic enzyme, which is activated synergistically by Sox9 and Nr5a1 [143]. In XY gonads, male-specific expression of Cyp26b1 mediates degradation of retinoic acid (RA), which inhibits germ cells from entering meiotic division by preventing exposure to RA [144, 145].

In XX gonads, progenitor cells from the coelomic epithelium show no obvious fate restriction and are unlikely to contribute to the supporting cell lineage (granulosa cells) at the embryonic stage [27, 29]. Instead, during the perinatal and early postnatal periods, coelomic epithelial cells ingress to the ovarian cortex and give rise to granulosa cells [146]. Subsequently, there is formation of the primordial follicles in which a single layer of granulosa cells completely surrounds and nurtures individual germ cells. In contrast to testis cord formation, follicular formation is critically dependent on the presence of germ cells [147, 148]. Specification of pre-granulosa cells begins in XX gonads, which is accompanied by ovarian-specific *Foxl2* expression at around E12.5 [149]. Repression of *Sox9* by the ovary-specific WNT signaling activity enables *Foxl2* upregulation in the supporting cell lineage of XX gonads.

#### Endocrine cell lineages (Leydig and theca cells)

In XY gonads, interstitial Leydig cells are derived from the coelomic epithelium and gonad–mesonephros border cells [150]. Early differentiation and expansion of the fetal Leydig cell lineage are regulated by Sertoli cells. For example, signaling activity of Desert Hedgehog (Dhh, also known as Patched 1), which is expressed in Sertoli cells, is required for specification of the fetal Leydig cell fate [151]. Signaling by the growth factor PdgfA, which is secreted from Sertoli cells, through its receptor Pdgfra in the interstitium is required for fetal and adult Leydig cell differentiation [152, 153]. In addition, the X-linked *aristaless*-related homeobox gene (*Arx*) is implicated in the regulation of Leydig cell differentiation [154]. Ectopic Nr5a1 upregulation in *Pod1* ^*lacZ/lacZ*^ gonads leads to a remarkable increase in the number of presumptive fetal Leydig cells [40], suggesting that Nr5a1 may contribute to fetal Leydig cell formation. Testosterone, the male sex steroid hormone, is synthesized by Leydig cells through the coordinated action of steroidogenic enzymes, many of which are regulated by Nr5a1. Subsequently, endocrine effects of the testosterone promote the differentiation of secondary male sexual characteristics of individuals. Testosterone, which functions through the androgen receptor (AR), masculinizes the rest of the body, including male-specific differentiation of the genital tract, external genitalia, and brain (for a review [26]). X-linked ATR-X (alpha thalassemia, mental retardation, X-linked) syndrome in males is characterized by mental retardation, facial dysmorphism, alpha thalassemia, and urogenital abnormalities including small testes. ATR-X modulates AR-dependent gene expression in spermatogenesis, which is important for the proliferation and survival of fetal Sertoli cells [155]. Fetal Leydig cells are also reported to produce a member of the TGF-β superfamily, activin A, which regulates Sertoli cell proliferation and fetal testis cord expansion [156].

In XX gonads, when the follicle has two layers of granulosa cells, theca cells are formed and localize to the outer surface of the follicle. Theca cells are derived from mesenchymal precursor cells in the ovarian stroma adjacent to the developing follicles. Currently, the factors that regulate theca cell differentiation are unknown. In association with ovarian follicles, theca cells play crucial roles in supplying sex steroid hormones required for oocyte development and physiological homeostasis of the body (for a review [157]).

#### Other cell lineages

After E11.5, a second population of somatic cells from the neighboring mesonephros migrates into the XY, but not XX gonad [158]. The migrated mesonephric cells in the testis are required to form and pattern the testis cords. Recent findings suggest that this cell population becomes endothelial cells exclusively and is incapable of differentiation into Sertoli cells [159, 160]. These endothelial cells contribute to vascular network formation in the XY gonads. The interstitium of the XY gonad also contains other uncharacterized cell types. For example, a cell population positive for the soluble integrin-binding protein Mfge8 is specifically localized to the border region between the gonads and mesonephros of the E10.0 coelomic epithelium. Subsequently, the Mfge8-positive cells expand around the border region and contribute to a previously uncharacterized somatic cell type that is distinct from Sertoli cells, Leydig cells, peritubular myoid cells, and the endothelial cells [161].

## Functional interaction between somatic cells and germ cells in the gonad

### Initiation of germ cell sexual differentiation

The gonad is an essential organ for differentiation of germ cells into mature gametes in both sexes, which are required to produce the next generation. Supporting Sertoli and granulosa cells interact with and nurture the germ cells. In testes, germ cells differentiate into sperms, whereas in ovaries, germ cells differentiate into oocytes.

PGCs settle into the genital ridge and interact with gonadal somatic cells at around E10.0 before sex determination occurs. Thereafter, male-specific RA degradation by Cyp26b1 prevents germ cells from entering meiotic division in XY gonads, but not in the XX gonad at around E13.5 [144, 145]. The interaction with gonadal somatic cells is considered to facilitate germ cell differentiation in which PGCs in the gonads exit their pluripotent and migratory states, and acquire competence to initiate sexual differentiation and enter meiosis. For example, PGCs in gonads start expressing germ cell-specific genes, such as genes encoding the RNA-binding protein *dazl* (deleted in azoospermia-like) and RNA helicase *mvh* (mouse vasa homolog, also known as *Ddx4*). Moreover, co-culture of embryonic germ (EG) cells with gonadal somatic cells induces *mvh* upregulation. *dazl* and *mvh* are essential for germ cell development in adult testes and important for gonadal germ cell development [162–169]. On the other hand, expression of pluripotency-related genes, such as *Pou5f1* (also known as *oct*-*3/4*) and *Alpl* [also known as *Akp2* encoding tissue non-specific alkaline phosphatase (TNAP)], is gradually decreased in gonadal PGCs. Recent whole-genome bisulfite sequencing has also shown that global loss of DNA methylation occurs in migratory PGCs, but some resistant regions become demethylated in PGCs only after they colonize the gonads [170]. These findings suggest that the interaction with gonadal somatic cells facilitates the initiation of sexual differentiation of germ cells, but its precise regulatory mechanisms remain to be elucidated.

Recently, PGC-like cells (PGCLCs) have been derived from mouse embryonic stem cells (ESCs) or inducible pluripotent stem (iPS) cells in vitro, which are capable of generating a live organism in both sexes [171–173]. However, the generation of functional gametes from PGCLCs requires the microenvironment of gonadal somatic cells. To generate functional sperms, XY PGCLCs can be injected into neonatal testes [172], whereas oocyte generation from XX PGCLCs requires co-culture with female gonadal somatic cells [171]. On the other hand, Buganim et al. [174] have generated induced embryonic Sertoli-like cells (ieSCs) by direct reprogramming of mouse embryonic fibroblasts (MEFs). Concomitant expression of five transcription factors, *Nr5a1, Wt1, Gata4, Sox9*, and *Dmrt1*, efficiently reprograms MEFs into ieSCs. These ieSCs facilitate germ cell survival in culture and contribute to the Sertoli cell population in vivo [174]. Such induced cells may be useful materials not only to perform biochemical studies of Sertoli cell differentiation, but also to establish in vitro gametogenesis systems. Because in vitro generation of fertile sperm is possible in cultured neonatal mouse testes [175], it is worthwhile testing the use of induced cells instead of endogenous cells.

### Plasticity of male and female supporting cells

Recently, the plasticity of the fate of male and female supporting cells in adult gonads has been reported in mice. Conditional inactivation of *Foxl2* in adult ovaries results in transdifferentiation of granulosa cells to Sertoli cells, which is accompanied by upregulation of some testicular genes including *Sox9* [176]. Furthermore, the reciprocal transdifferentiation of Sertoli cells to granulosa cells is found in adult mouse testes with conditional inactivation by *Nr5a1*-*Cre* or *Dhh*-*Cre* of a member of the DM domain transcription factor family, *Dmrt1* [177]. However, loss of either *Foxl2* or *Dmrt1* in embryonic gonads does not impair sex determination or differentiation of gonads until the perinatal stage [178–180]. Therefore, these findings suggest that distinct mechanisms may control the maintenance of the supporting cell fate in adult mouse gonads and the determination of the supporting cell fate when sex determination occurs in embryonic gonads.

The forkhead transcription factor *Foxl2*, the HMG transcription factor *Sox9*, and the DM domain transcription factor *Dmrt1* are known to be evolutionally conserved among animal species in terms of gene structure, expression pattern, and their functions in sex determination (for a review [181]). Manipulation of these evolutionally conserved factors achieves postnatal cell fate reprogramming of the supporting cells in mouse adult gonads. Compared with ovarian-specific *Foxl2* and testicular-specific *Sox9*, homologs of *Dmrt1* occasionally show opposing functions in sex determination among animal species. For example, the Y-linked DM gene *DMY* acts as the testis-determining gene in some *Medaka* fish species [182], whereas W-linked *DM*-*W* promotes ovarian development in *Xenopus laevis* [183]. ChIP assays of adult mouse testes have demonstrated that Dmrt1 directly binds to the regulatory regions of testicular genes [i.e., *Sox8*, *Sox9*, and *the* Ptgds receptor (*Ptgdr*)] and ovarian genes [*Foxl2*, *Wnt4,* *R*-*spondin1*, and the estrogen receptor (*Esr*)] [177]. Therefore, Dmrt1 may regulate the expression of both testicular and ovarian genes to maintain the Sertoli cell fate in adult mouse testes. In terms of regulating both testicular and ovarian genes, Dmrt1 functions appear to be partially conserved among animal species.

In contrast to the transdifferentiation of postnatal supporting cells, sex reversal of germ cells is unlikely to occur after sex determination, even in the atypical gonadal environment of mice. Recently, only two genes on the Y chromosome, the testis determinant factor *Sry* and spermatogonial proliferation factor *Eif2s3y*, have been shown to enable differentiation of XX germ cells into a round spermatid-like cell type in the testes, which can give rise to the next generation by injection into an oocyte [184]. Further investigations will be needed to address the plasticity of male and female germ cells in gonads.

## Conclusions and prospects

The bipotential genital ridge is an essential organ for sex determination of individuals. Nr5a1 is a key transcriptional factor in the formation and development of genital ridges. The formation of the long and narrow genital ridge begins from the anterior part of the coelomic epithelium, and Gata4, Six1, and Six4 contribute to *Nr5a1* expression in the progenitor cells (Fig. 1). A proportion of these cells give rise to *Sry*-expressing Sertoli cells in XY gonads. Despite identification of *Sry* as the testis-determining gene of mammals in 1990, mechanisms underlying the strictly controlled expression of *Sry* and its functions in sex determination are largely unknown. Recent findings have revealed that transcriptional networks and histone modification govern *Sry* upregulation (Fig. 3). Sry primes initial upregulation and subsequent maintenance of *Sox9* expression at a high level for testis determination. It has been suggested that appropriate timing and a sufficient level of *Sry* expression and an appropriate number of *Sry*-expressing cells in the genital ridge are crucial for maintenance of the high level of *Sox9* expression to promote testicular differentiation.

During the past few decades, we have gained considerable knowledge of the regulatory gene network in testicular differentiation by identification of key factors. However, these findings still cannot fully explain some cases of DSD. It is likely that the sexual fate decision in the developing gonad depends on a complex network of interacting factors. Further comprehensive approaches will be required to elucidate the regulatory gene network that governs testicular differentiation more precisely.

By employing stem cell biology approaches, germ cells (PGCLCs) have been derived from ESCs and iPS cells, and supporting cells in male gonads (ieSCs) have been generated by direct reprogramming. Induction of other cell lineages including supporting cells in female gonads will be helpful to further elucidate the functional interaction between somatic cells and germ cells in gonads. Such induced cells may be useful materials not only to perform biochemical studies, but also to establish in vitro gametogenesis systems for both sexes. Furthermore, a combination of cell-based analyses of these induced cells and genetic studies in mouse models will significantly contribute to understanding the causes of unexplained DSD in human patients.

## Box 1

### Genes involved in genital ridge formation

#### Nr5a1

Orphan nuclear receptor adrenal 4-binding protein (*Ad4BP*) [also known as nuclear receptor subfamily 5, group A, member 1 (*Nr5a1*) and steroidogenic factor 1 (*Sf1*)] was first identified as a gene encoding a common transactivating factor that binds to the promoter region of steroid hydroxylase genes [52–54]. In mice, inactivation of *Nr5a1* *(Nr5a1* ^−*/*−^ *)* leads to complete agenesis of the gonad and adrenal gland [48–50], whereas human *NR5A1* inactivation results in decompensating primary adrenal failure and small intra-abdominal gonads [185].

#### Wt1

The gene encoding the zinc finger transcription factor Wilms’ tumor 1 (*Wt1*) was first identified as the responsible gene for Wilms’ tumors. In human patients, mutations of *WT1* cause a form of kidney cancer primarily in children [186]. Wt1 has two isoforms with or without an additional three amino acids (lysine, threonine, and serine) between the third and fourth zinc finger (+KTS and −KTS, respectively). These two isoforms play distinct roles during embryogenesis. *Wt1−KTS* is essential for the formation and development of the bipotential gonad [32].

#### Lhx9

LIM homeobox 9 (Lhx9) is a member of the LIM homeobox protein family. Inactivation of *Lhx9* results in regression of the gonads by E13.5 due to markedly reduced cell proliferation in the bipotential gonads [30].

#### Emx2

*Empty spiracles* homeobox 2 (*Emx2*) is a homolog of the *Drosophila* head gap gene *empty spiracles* (*ems*). Mouse embryos lacking *Emx2* (*Emx2* ^−*/*−^) have agenesis of the kidneys, ureters, gonads, and genital tracts, while the adrenal glands and bladder develop normally [187]. In forming *Emx2* ^−*/*−^ genital ridges, coelomic epithelial cells lose their cellular polarity, which is accompanied by aberrant tight junction assembly. There is also an apparent decrease in the number of migrated *Emx2* ^−*/*−^ coelomic epithelial cells in the mesenchymal compartment, resulting in impaired gonad formation [28].

#### *Six1* and *Six4*

*Six1* and *Six4* genes belong to the mammalian homolog of the *Drosophila* *sine oculis homeobox* (*Six*) family, which includes six member genes (*Six1* to *Six6*) in the mouse genome. These genes encode transcriptional factors with characteristic Six- and homeo-domains. Six1 and Six4 perform redundant functions in mouse embryogenesis. *Six1* and *Six4* are located in the same genomic region (within 100 kb on chromosome 12) and have highly overlapping tissue expression during mouse embryogenesis (for reviews [43, 44]). Six1 and Six4 bind to a common binding site (MEF3 site) and transactivate genes belonging to the myogenic regulatory factor family [188–190]. *Six1* and *Six4* double-mutant (*Six1* ^−*/*−^ *; Six4* ^−*/*−^) mouse embryos are phenotypically different from *Six1* ^−*/*−^ to *Six4* ^−*/*−^ single-mutant mouse embryos, indicating redundant functions of Six1 and Six4 in mouse embryonic development. For example, loss of both *Six1* and *Six4*, but not alone, leads to a reduction of gonadal size in both sexes and impaired testicular differentiation in XY gonads. Our previous study further demonstrated that Six1 and Six4 play an essential role in size determination of the mouse gonad by regulating the initial growth of gonadal precursor cells before the onset of *Sry* expression [42].

#### Gata4

GATA-binding protein 4 (Gata4) is a member of the GATA zinc finger transcription factor family. Mouse embryos with conventional inactivation of *Gata4* (*Gata4* ^−*/*−^) die before the genital ridge forms [191, 192]. Mouse embryos that are conditionally deficient for *Gata4* after E8.75 show no signs of the initiation of genital ridge formation, because their coelomic epithelium remains as a morphologically undifferentiated monolayer [34]. In XY mouse embryos that are homozygous for a *Gata4* knock-in allele (*Gata4* ^*ki*^), which abrogates Gata4 binding to the cofactor Fog2 (also known as Zfpm2), the genital ridges form but further differentiation into testes is blocked [98].

#### Pod1

Embryos lacking the basic helix–loop–helix transcription factor *Pod1* (*Pod1* ^*lacZ/lacZ*^) (also known as *Tcf21/bHLHa23/capsulin/epicardin*) show hypoplastic development in both XX and XY gonads [40]. Impaired growth begins at E11.5 in both XX and XY genital ridges with slight shortening of their length and an irregular surface. *Pod1* ^*lacZ/lacZ*^ gonads further show defects in the formation of testis cords and testis-specific coelomic blood vessels, and a remarkable increase in the number of presumptive fetal Leydig cells that express the cholesterol side-chain cleavage enzyme [40].

#### Cbx2

In addition to the transcriptional factors, the chromatin modification and remodeling factor M33 (also known as Cbx2) is involved in the formation and development of the genital ridges. In genital ridges lacking *Cbx2* (*Cbx2* ^−*/*−^), the proliferation and ingression of coelomic epithelial cells are likely to be normal, but the gonadal cells show defective proliferation at later stages [35, 36].

#### Insulin/IGF signaling pathway

Constitutive ablation of the insulin/insulin-like growth factor (IGF) signaling pathway also leads to impaired gonadal development. Mouse embryos lacking the insulin receptor (*Insr*) and IGF receptor 1(*Igf1r*) exhibit reduced proliferation rates of somatic progenitor cells in both XX and XY gonads prior to sex determination together with complete agenesis of the adrenal gland and the absence of testis development [38, 39]. Ablation of insulin/IGF signaling activity also leads to the male-to-female sex reversal accompanied by reduced *Sry* expression. However, it is thought that reduced *Sry* expression could be a secondary effect of the general proliferation defect and subsequent reduction of *Sry*-expressing pre-Sertoli cells, rather than its direct effect on the regulation of *Sry* expression.

## Box 2

### Genes involved in *Sry* expression

#### Wt1

The Wt1−KTS isoform binds to the *SRY* promoter region and transactivates SRY expression in vitro [93]. In addition, Wt1−KTS binds to the *Nr5a1* promoter sequence [33]. Specific abolition of *Wt1−KTS* in mouse embryos results in smaller gonad formation because of increased apoptosis. However, testicular differentiation markers such as *Sox9* and *MIS* are detected in a small cluster of cells [32]. On the other hand, abolition of the *Wt1*+*KTS* isoform results in reduced *Sry* levels and a sex-reversal phenotype [32], although Wt1+KTS does not transactivate the *Sry* promoter in vitro [93, 96]. Wt1+KTS is reported to function at the post-transcriptional level to regulate RNA expression and to promote translation of an unspliced RNA [97]. Then Wt1+KTS is considered to be implicated in the post-transcriptional regulation of *Sry* mRNA in testis determination. Abolition of the *Wt1*+*KTS* isoform also results in a decrease in cell proliferation of Sry-expressing cells in the coelomic epithelium, which is rescued by the addition of exogenous FGF9 to the cultured gonad [112]. In addition, we previously reported that stable trasfection of *Sry* into XX embryonic stem cells (ESCs), but not into gonadal somatic cell line cells (i.e., M15 and TM-4 cells), results in the upregulation of *Wt1* [193].

#### Nr5a1

*NR5A1* haploinsufficiency in humans causes a male-to-female sex-reversal phenotype [185, 194–197]. An effect of *Nr5a1* haploinsufficiency on testicular differentiation is found in *Nr5a1* ^+*/*−^ B6 XY^AKR^ mice [198], but usually leads to normal testis development in mice. In *Nr5a1* ^−*/*−^ mouse embryos, both XX and XY gonads regress by E12.5 [48–50]. Because no *Sry* expression is observed in *Nr5a1* ^−*/*−^ gonads, it has been proposed that Nr5a1 may be one of the upstream regulators of *Sry* (for a review, [24] ).

#### *Fog2* and *Gata4*

Embryos lacking Friend of GATA-2 (*Fog2*, also known as zinc finger protein, multitype 2, *Zfpm2*) show reduced *Sry* expression and a sex-reversal phenotype [98]. Conventional inactivation of *Gata4* in mouse embryos causes embryonic death at around E7.0–E9.5 due to abnormalities in ventral morphogenesis and heart tube formation before genital ridge formation [191, 192]. However, mice containing homozygous mutant alleles of *Gata4* ^*ki*^, which abrogate the interaction of Gata4 with Fog, also show reduced *Sry* expression and a sex-reversal phenotype [98]. These findings suggest that the interaction of Gata4 and co-factor Fog2 is critical for *Sry* activation.

#### *Six1* and *Six4*

Previously, we find that Six1 and Six4 play crucial roles in sex determination by upregulating *Sry* expression. XY *Six1* ^−*/*−^; *Six4* ^−*/*−^ gonads fail to upregulate *Sry* expression and show impaired testicular differentiation. We further identified *Fog2* as a direct target of Six1 and Six4, which is considered necessary for upregulation of *Sry*. Therefore, the Six1/Six4-Fog2 pathway is required for *Sry* upregulation. In addition, *Six1* ^−*/*−^ *;Six4* ^−*/*−^ genital ridges show few Sry-expressing cells in the coelomic epithelium region [42]. This observation suggests that migration of gonadal progenitor cells through the basement membrane layer is unlikely to be essential for *Sry* activation.

#### Map3k4

The boygirl (*byg*) mutation, which causes XY gonadal sex reversal, is an A to T transversion that introduces a premature stop codon in the gene encoding mitogen-activated protein kinase (*Mapk*) kinase kinase *Map3k4* (also known as *Mekk4*) [99]. E11.5 *byg/byg* gonads show a growth deficit and dramatic reduction of *Sry*. MKK4, a direct target of MAP3K4 and p38 MAPK, is activated in the coelomic region of the E11.5 XY wild-type gonad, suggesting that MAPK signaling may be involved in gonadal somatic cell growth and regulation of *Sry* expression [99].

#### Gadd45g

Mice lacking *Gadd45g* (*Gadd45g* ^−*/*−^), which was identified as a gene upregulated by agents that cause DNA damage, also show XY gonadal sex reversal caused by disruption of *Sry* expression [101, 102]. *Gadd45g* and *Map3k4* genetically interact during sex determination, and transgenic overexpression of *Map3k4* rescues gonadal defects in *Gadd45g* ^−*/*−^ embryos. *Sry* expression is delayed and reduced in *Gadd45g* ^−*/*−^ gonads. The sex-reversal phenotype of *Gadd45g* ^−*/*−^ embryos is associated with reduced phosphorylation of p38 MAPK and Gata4 [101, 102].

#### Cbx2

Mutations of the polycomb group gene *Cbx2* cause XY sex reversal in both mice and humans [35, 199]. *Cbx2* ^−*/*−^ mouse embryos show reduced *Sry* expression, and growth defects are observed in the genital ridge as soon as *Sry* expression begins [35]. Either *Sry* or *Sox9* transgenes can rescue the impaired testicular differentiation in *Cbx2* ^−*/*−^ mouse embryos [36]. Direct binding of Cdx2 to the *Nr5a1* locus shown by ChIP assays and reduced *Nr5a1* expression in *Cbx2* ^−*/*−^ gonads suggest that Cbx2 regulates *Nr5a1* expression [37]. However, a genetic interaction between *Cbx2* and *Sry* is unclear.

#### Jmjd1a

Kuroki et al. [104] reported male-to-female sex reversal in mice lacking the H3K9 demethylase *Jmjd1a* (also known as *Tsga/Jhdm2a/Kdm3a*) (*Jmjd1a* ^−*/*−^), which is accompanied by reduced expression of *Sry*. *Jmjd1a* ^−*/*−^ mice show abnormal sex differentiation depending on the genetic background. Jmjd1a is expressed in gonadal somatic and germ cells, but not in mesonephric cells at E11.5. *Jmjd1a* has the highest expression level among genes encoding enzymes involved in the maintenance of H3K9 methylation in E11.5 gonadal somatic cells. *Jmjd1a* expression increases from E10.5 and reaches a peak at around E11.5. ChIP assays using purified Nr5a1-positive gonadal somatic cells from E11.5 gonads show that Jmjd1a directly binds to regulatory regions within the *Sry* locus. Furthermore, inactivation of *Jmjd1a* leads to a significant increase in H3K9me2 levels within the *Sry* locus without changing histone H3 occupancy and H3K9me2 levels of the *Sox9* locus [104].

## Abbreviations

Alpl/Akp2/TNAP: Alkaline phosphatase, liver/bone/kidney
AR: Androgen receptor
Arx: X-linked *aristaless*-related homeobox
ATR-X: Alpha thalassemia, mental retardation, X-linked
Cbln4: Cerebellin precursor 4
Cbx2/M33: Chromobox homolog 2/mouse polycomb group member M33
ChIP: Chromatin immunoprecipitation
Col9a3: Collagen, type IX, alpha 3
Cyp17a1: Cytochrome P450, family 17, subfamily a, polypeptide 1
Cyp26b1: Cytochrome P450, family 26, subfamily b, polypeptide 1
Dax1/Nr0b1: Dosage-sensitive sex reversal, adrenal hypoplasia critical region, on chromosome X, gene 1/nuclear receptor subfamily 0 group B, member 1
dazl: Deleted in azoospermia-like
Dhh/Ptch1: Desert hedgehog/patched 1
Dmrt1: *Doublesex* and *mab*-*3* related transcription factor 1
DSD: Disorders of sex development
E: Embryonic day
EG cells: Embryonic germ cells
EGF: Epidermal growth factor
Eif2s3y: Eukaryotic translation initiation factor 2, subunit 3, structural gene Y-linked
EMT: Epithelial mesenchymal transition
Emx2: *Empty spiracles* homeobox 2
FGF9: Fibroblast growth factor 9
FGFR2: Fibroblast growth factor receptor 2
ESCs: Embryonic stem cells
Esr: Estrogen receptor
Fog2/Zfpm2: Friend of GATA-2/zinc finger protein, multitype 2
Foxl2: Forkhead box L2
Gadd45g: Growth arrest and DNA damage-inducible 45 gamma
Gata4: GATA binding protein 4
HMG: High mobility group
3β-Hsd: 3 Beta-hydroxysteroid dehydrogenase
ieSCs: Induced embryonic Sertoli-like cells
Ifitm3/mil-1/fragilis: Interferon induced transmembrane protein 3/mouse Ifitm-like protein-1/fragilis
IGF: Insulin-like growth factor
Jmjd1a/Tsga/Jhdm2a/Kdm3a: Jumonji domain-containing protein 1A/testis-specific gene A/jmjC domain-containing histone demethylation protein 2A/lysine (K)-specific demethylase 3A
iPS: Inducible pluripotent cells
lacZ: Beta-D-galactosidase
Lhx9: LIM homeobox 9
MAP3K4/MEKK4: Mitogen-activated protein kinase kinase kinase 4
MAPK: Mitogen-activated protein kinase
MEFs: Mouse embryonic fibroblasts
Mfge8: Milk fat globule-EGF factor 8
MKK4: Mitogen-activated protein kinase kinase 4
MIS/Amh: Müllerian inhibitory substance/anti-Müllerian hormone
mvh/Ddx4: Mouse vasa homolog/DEAD box polypeptide 4
Nr5a1/Ad4BP/Sf1: Nuclear receptor subfamily 5, group A, member 1/adrenal 4 binding-protein/steroidogenic factor 1
Ntf3: Neurotrophin 3
oct-3/oct-4/Pou5f1: Octamer-binding transcription factor 3/octamer-binding transcription factor 4/POU domain, class 5, transcription factor 1
PdgfA: Platelet-derived growth factor subunit A
Pdgfra: Platelet-derived growth factor receptor alpha
Pod1/Tcf21/bHLHa23/capsulin/epicardin: Podocyte-expressed 1/transcription factor 21/class A basic helix–loop–helix protein 23/capsulin/epicardin
PGCs: Primordial germ cells
PGCLCs: PGC-like cells
Prdm1/Blimp1: PR domain-containing 1, with ZNF domain/B-lymphocyte-induced maturation protein 1
Ptgds: Prostaglandin D2 synthase
Ptgdr: Prostaglandin D2 receptor
RA: Retinoic acid
Rspo1: Roof plate-specific Spondin 1 (R-spondin 1)
Six1, 4: *Sine oculis*-related homeobox 1, 4
Sox3, 8, 9, 10: Sry-related HMG box 2, 8, 9, 10
SP1: Specificity protein 1
Sry: Sex determination region on Y chromosome
*Tes*: Testis-specific enhancer region of *Sox9*
TGF-β: Transforming growth factor-β
Wnt4: Wingless-type MMTV integration
Wt1: Wilms’ tumor 1
Wt1+KTS: Isoform of Wt1 containing an additional three amino acids (lysine, threonine, and serine)
Wt1−KTS: Isoform of Wt1 not containing an additional three amino acids (lysine, threonine, and serine)
